# Characterization of Transposon-Derived Accessible Chromatin Regions in Rice (*Oryza Sativa*)

**DOI:** 10.3390/ijms23168947

**Published:** 2022-08-11

**Authors:** Aicen Zhang, Wenli Zhang

**Affiliations:** State Key Laboratory for Crop Genetics and Germplasm Enhancement, Collaborative Innovation Center for Modern Crop Production Co-sponsored by Province and Ministry (CIC-MCP), Nanjing Agricultural University, No.1 Weigang, Nanjing 210095, China

**Keywords:** transposable elements, accessible chromatin regions, regulation of gene transcription, *Oryza sativa*

## Abstract

Growing evidence indicates that transposons or transposable elements (TEs)-derived accessible chromatin regions (ACRs) play essential roles in multiple biological processes by interacting with *trans*-acting factors. However, the function of TE-derived ACRs in the regulation of gene expression in the rice genome has not been well characterized. In this study, we examined the chromatin dynamics in six types of rice tissues and found that ~8% of ACRs were derived from TEs and exhibited distinct levels of accessibility and conservation as compared to those without TEs. TEs exhibited a TE subtype-dependent impact on ACR formation, which can be mediated by changes in the underlying DNA methylation levels. Moreover, we found that tissue-specific TE-derived ACRs might function in the tissue development through the modulation of nearby gene expression. Interestingly, many genes in domestication sweeps were found to overlap with TE-derived ACRs, suggesting their potential functions in the rice domestication. In addition, we found that the expression divergence of 1070 duplicate gene pairs were associated with TE-derived ACRs and had distinct distributions of TEs and ACRs around the transcription start sites (TSSs), which may experience different selection pressures. Thus, our study provides some insights into the biological implications of TE-derived ACRs in the rice genome. Our results imply that these ACRs are likely involved in the regulation of tissue development, rice domestication and functional divergence of duplicated genes.

## 1. Introduction

Transposable elements (TEs) have been the subject of much debate since they were first discovered and described as “controlling elements” in maize (*Zea mays*) by Barbara McClintock [1]. They represent a major type of repetitive DNA sequences and account for large proportions of many plant genomes, such as 40% in rice, 60% in cotton, and more than 80% in wheat and maize [2,3,4,5]. Initially, TEs were considered as foreign DNA invading the host genome, and their genome-wide propagation, or so-called transposition, frequently occurs during evolution or in response to biotic and abiotic stresses [6,7,8,9,10]. According to their transposition mechanisms, TEs are usually divided into two distinct classes [11]. Class I TEs transpose via a “copy and paste” mechanism with a reversely transcribed RNA as an intermediate. They can be further classified into several subtypes with distinct DNA sequence features, including long terminal repeat (LTR) elements such as the *Gypsy* superfamily and the *Copia* superfamily, long interspersed nuclear elements (LINEs), and short interspersed nuclear elements (SINEs). The Class II TEs, referred to as DNA transposons, transpose via a “cut and paste” mechanism with a DNA as an intermediate, they can be further grouped into several superfamilies based on their transposase proteins and sequence structures, such as *CACTA*, *Mutator*, *Tc1/Mariner*, et al. In addition, a new type of DNA transposon, *Helitron*, can transpose via the “rolling circle replication” model [11]; another type of complex Class II DNA transposons, called *Polintons,* transposing in a self-synthesizing manner has been found in several eukaryotic species, like protists, fungi, and animals [12].

The jumping of TEs frequently disrupts genome structures, resulting in genome instability, and induces some deleterious mutations in the host genome [13]. The host genomes have evolved multiple mechanisms to regulate TE transposition, including enzymatic systems for elimination of TEs and epigenetic mechanisms for silencing TE activities, such as DNA methylation, histone modifications, and RNA interference (RNAi) [14,15,16,17]. However, accumulating evidence has shown that a subset of TEs can be activated under certain conditions, such as in a specific tissue or developmental stage or in responses to external stresses, and they can play vital roles in the regulation of gene transcription, including generation of novel or abnormal transcripts, formation of novel *cis*-regulatory elements (CREs) such as alternative promoters and enhancers essential for the regulation of gene transcription, and alteration of chromatin features of nearby genes, thereby being involved in various biological processes [18,19]. These findings indicate that TE transcription leads to complex outcomes in the host genome.

Open chromatin, also referred to as accessible chromatin, represents unique chromatin regions with less nucleosome occupancy or nucleosome depletion (nucleosome-free regions) [20]. It usually harbors intricate CREs, such as promoters, enhancers, silencers, and insulators, which are essential for the modulation of gene transcription through interactions with various *trans*-acting factors during growth and development and in response to environmental cues in mammals and plants [21,22]. Open chromatin has been extensively investigated in eukaryotic genomes, including yeast, mammals, humans, and plants [23,24,25,26,27]. For instance, chromatin openness exhibits a positive correlation with the expression of corresponding genes; 25% of open chromatin sites are located in the promoters of rice genes [23]. Moreover, distal regulatory elements (DREs) in open chromatin regions have been found to be dynamic across different tissues and associated with tissue-specific gene expression in rice [28].

Accumulating evidence has shown that TEs can potentially contribute to the formation of open chromatin owing to partially/fully overlapping open chromatin in mammalian and plant genomes [26,29,30,31]. Forty-four percent of open chromatin regions are associated with TEs in human cells [29]. Over 20% of TEs overlapping open chromatin are potentially related to tissue development in mouse [31]. Similarly, TE-derived open chromatin has been reported to regulate gene transcription [30], and three TE families overlapping DNase Ⅰ hypersensitive sites (DHSs) can function as enhancer candidates in maize [26]. Some TEs located in the distal regions of genes contain most of the TF binding sites (TFBSs) in wheat, and their expansion is associated with the responses of wheat-specific genes to environmental stimuli [32].

However, relationships between TEs and accessible chromatin regions (ACRs) haven’t been well characterized in rice as compared with other plants like maize. To this end, we analyzed the dynamics of chromatin accessibility in six rice tissues, associated accessible chromatin regions (ACRs) with different TE superfamilies, and characterized the genomic features of corresponding TEs and ACRs in rice. We also investigated the role of TE-derived ACRs in the regulation of tissue-specific and duplicated gene expression. Our study provides some insights into the contribution of TEs in ACRs formation, thereby advancing our understanding of the biological implications of TE-derived ACRs in the rice genome.

## 2. Results

### 2.1. Identification of TE-Related Accessible Chromatin Regions in Rice

**Figure 1:**
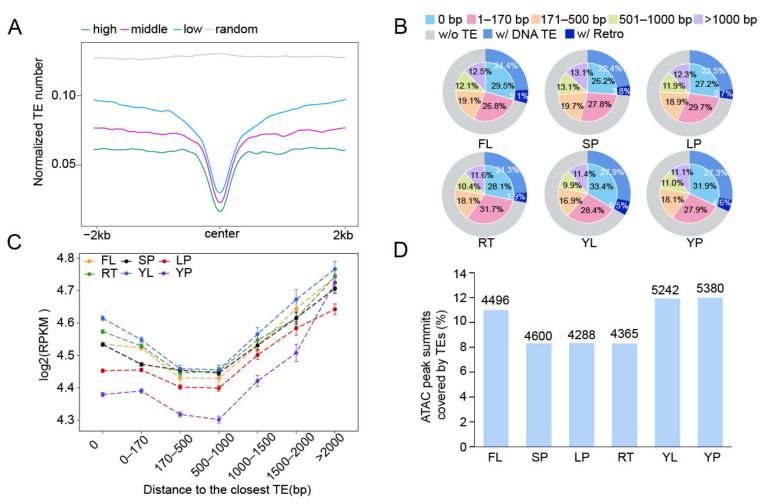
The TE-overlapping ACRs (accessible chromatin regions) in rice. (**A**) Curve plot showing normalized TE number across ±2 kb of ACR center (in flag leaf). All ACRs were divided into three subgroups (high, mid, low) according to their levels of accessibility. The same number of randomly selected regions were used as control. (**B**) Pie plot showing the ratio of ACRs with different distances to the nearest TEs (0 bp, representing ACRs with at least 1 bp overlap with TEs, 1~170 bp, 171~500 bp, 501~1000 bp, >1000 bp) in six tissues (FL, flag leaf; SP, stamen & pistil; LP, lemma & palea; RT, root; YL, young leaf; YP, young panicle), 26.2% (SP) to 33.4% (YL) ACRs had at least 1 bp overlap with TEs (*w/o* represents “without”, *w/* represents “with”). (**C**) The accessibility of ACRs with different distances to the nearest TEs in six tissues, the reads of ATAC-seq within ACRs were normalized by reads per kilobase per million mapped reads (RPKM). (**D**) Bar plots illustrating the ratio and number of ACRs with their summits covered by TEs.

To identify TE-related accessible chromatin regions in rice, we used the published ATAC-seq (Assay for Transposase-Accessible Chromatin with high throughput sequencing) data derived from six tissues [33], including young leaf (YL), flag leaf (FL), root (RT), stamen & pistil (SP), lemma & palea (LP), and young panicle (YP). The ATAC-seq is a robust technique widely applied for global profiling of chromatin accessibility through sequencing DNA fragments preferentially tagged by Tn5 transposase dimer. To minimize the impact of sequencing depth on peak calling, we down-sampled ATAC reads to the same amount for each tissue, and identified 43,988, 40,899, 52,601, 55,363, 51,395, and 44,884 ACRs corresponding to YL, FL, RT, SP, LP, and YP, respectively (Figure S1). All ACRs were further divided into three subgroups, high, middle, and low accessibility, according to the density of normalized read counts (RPKM value, reads per kilobase per million mapped reads), reflecting the degree of chromatin accessibility. We then counted TE number across ±2 kb of ACRs with different accessibility levels, and found that the normalized TE number was negatively associated with the level of chromatin accessibility in each tissue, even though the overall chromatin openness around TEs was dramatically lower than random (Figure 1A and Figure S2). After a closer examination, we found that the percentage of ACRs with at least 1 bp overlapping TEs ranged from 26.2% in SP to 33.4% in YL. Surprisingly, we found that nearly 90% of the ACRs were located within 1 kb of the nearest TEs (Figure 1B) and exhibited less variations in the chromatin accessibility levels. In contrast, the chromatin accessibility of ACRs located more than 1 kb away from the nearby TEs increased with increasing distance to nearby TEs (Figure 1C), indicating that TE presence does not favor chromatin openness. After looking into TE types, we found that the majority of the TEs overlapping with ACRs were DNA transposons (24.4~27.9%) and the rest (3.7~5.5%) were retrotransposons (Figure 1B).

Next, we calculated the coverage ratio of ACRs by TE sequences and found that only a small proportion of ACRs were fully covered by TEs (Figure S3). For instance, DNA transposons *hAT* and *Helitron* covered ca. 25% of the full ACR length (the length of ATAC peak) on average, while *PIF_Harbinger* and *Tcl_Mariner* exhibited the least coverage on ACRs. This was possibly caused by the short length of their DNA sequences. Most *Gypsy* can fully cover the overlapping ACRs. These results showed that most ACRs covered the junctional regions of TE/non-TE DNA, which is similar to the findings in mouse [34]. A similar trend was observed for the coverage of the ATAC peak summits by the overlapping TEs (Figure 1D). There were 4288–5380 ACRs (accounting for 8.3~12.0% of all ACRs) with their summits overlapping with TEs (Figure 1D), and *Mutator*, *hAT*, and *Gypsy* exhibited more coverage of ACR summits than others (Figure S4).

Taken together, these results indicate that some TE sequences, in combination with their flanking non-TE DNA sequences, are able to contribute to the formation of ACRs, implying potential regulatory roles of TEs in rice.

### 2.2. Profiling of TEs in Proximal and Distal ACRs

**Figure 2:**
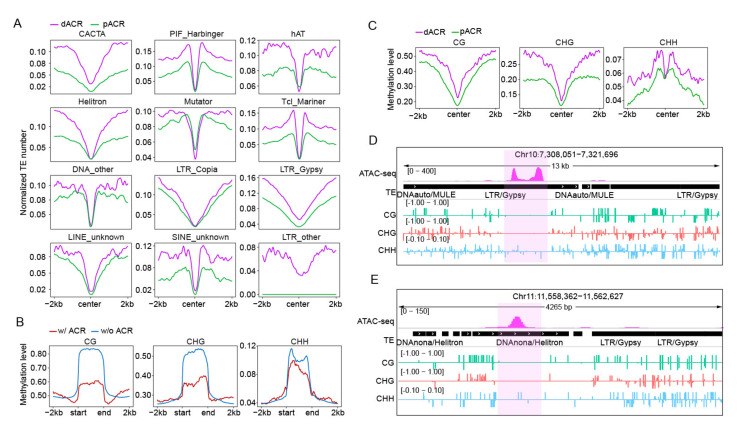
Distributions of TEs around ACRs and the methylation levels of corresponding ACRs and TEs. (**A**) Curve plots showing normalized TE number across ±2 kb of the center of pACRs (promoter ACRs) and dACRs (distal ACRs). (**B**) Average methylation level (CG, CHG, CHH) distribution over TEs with (*w*/) ACRs and without (*w*/*o*) ACRs. (**C**) The methylation level (CG, CHG, CHH) across ±2 kb of the center of TE-derived pACRs and dACRs. (**D**,**E**) IGV snapshots showing the methylation level around a *Copia* retrotransposon-derived ACR (**D**) and a *Helitron* DNA transposon-derived ACR (**E**), the ACRs were hypomethylated while the other regions located in TEs were hypermethylated.

It was reported that ACRs harbor *cis*-regulatory elements (CREs), which can be derived from TE sequences [21]. To explore whether TEs are also associated with functional ACRs in the rice genome, we merged ACRs from six tissues and obtained a total of 83,087 ACRs (Table S1). We then divided them into four subtypes according to their genomic loci, including 38,676 ACRs (46.5%) located within 2 kb upstream of the TSSs of genes (hereafter designated as promoter ACRs, pACRs); 14,622 ACRs (17.6%) located >2 kb from their nearest genes (hereafter designated as distal ACRs, dACRs); 16,814 ACRs (20.3%) overlapping with 2 kb downstream of the TTSs of the genes (hereafter designated as downstream ACRs, dnACRs); and the remaining 12,975 (15.6%) ACRs overlapping gene body regions (hereafter designated as genic ACRs, gACRs) (Figure S5). We then calculated the TE number across ±2 kb around the center of the pACRs and dACRs (Figure 2A). As shown in Figure 2A, the majority of TEs tended to be absent in the center of ACRs, and the dACRs exhibited a higher normalized TE number than the pACRs. Unlike other TE superfamilies, the short elements *PIF_Harbinger* and *Tcl_Mariner* were significantly enriched in the ACR boundary. And the number of *Mutator* and *hAT* was higher in the center region of pACRs compared with dACRs. (Figure 2A). These analyses showed that TEs exhibited a TE-type dependent distribution in or near the ACRs.

It is well known that TEs are hypermethylated, while open chromatin is hypomethylated [35], driving us to assess the DNA methylation levels of TE-derived ACRs. To this end, we analyzed public BS-seq data derived from young rice leaves [28]. After calculating the DNA methylation levels in each cytosine context in ±2 kb flank regions and bodies of related TEs, we observed that the TE body was hypermethylated relative to the flanking regions in CG, CHG and CHH context (Figure 2B), with the methylation level of ACR-related TEs showing a significant decrease than that of TEs without ACRs in TE bodies (Figure 2B). In addition, we also found that TEs with ACRs were significantly enriched with active histone mark (H3K4m3 and H3K27ac) in their bodies than that of TEs without ACRs, while the repressive mark H3K27me3 was more enriched in the flanking regions of TEs without ACRs, this further confirmed the relatively open state of TEs with ACRs (Figure S6). In contrast, the TE-derived ACRs were hypomethylated in their centers, and the CHH context was enriched in the region next to the center (Figure 2C), and the TE-derived dACRs showed a higher methylation level than pACRs. Corresponding to the methylation variations, we found the TE-derived pACRs were more open than that of dACRs (Figure S7). Thus, TEs exhibited an overall negative association between DNA methylation and accessible regions, TE-derived ACRs were prone to form in the hypomethylated regions within TEs. For instance, a *Copia* retrotransposon-derived ACR and a *Helitron* DNA transposon-derived ACR showed a hypomethylation level as compared to surrounding areas (Figure 2D,E). These results indicated that DNA methylation levels might be key determinants of the formation of TE-derived ACRs.

Taken together, these results demonstrate that the differential distributions of TE types around dACRs and pACRs may be caused by methylation changes in the underlying DNA sequences.

### 2.3. Involvement of TE-Derived ACRs in the Regulation of Tissue-Specific Gene Expression

**Figure 3:**
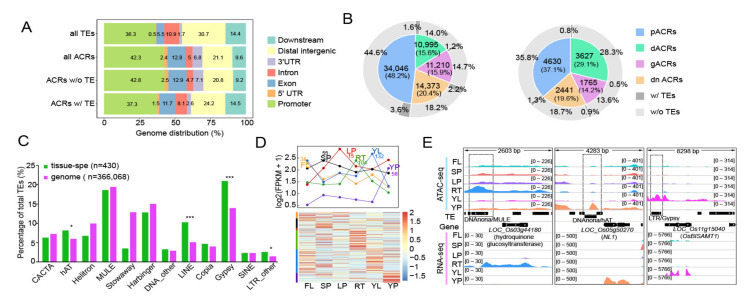
The association between TE-derived tissue-specific ACRs and tissue-specific gene expression.(**A**) Sub-genomic distributions of all TEs, all ACRs, ACRs without (*w*/*o*) TE and ACRs with (*w*/) TEs, including promoters (upstream 2 kb), exons, introns, downstream (2 kb) and distal intergenic regions. (**B**) Pie plot showing the ratio of different types of common (**left**) and tissue-specific ACRs (**right**) based on their distances to the nearest genes, 8.6% and 3.5% of these ACRs were TE-derived ACRs (>50% ACRs length covered by TE sequences), respectively. (**C**) Bar plot illustrating the percentage of TE superfamilies associated with tissue-specific ACRs and the percentage of TE superfamilies in the whole genome, significance test was determined using hypergeometric test, * *p* < 0.05, *** *p* < 0.001. (**D**) The expression pattern of the nearest genes of TE-derived tissue-specific ACRs, the lineplot (upper) showing the mean expression levels of genes in each tissue, each row in the heatmap (bottom) represents a gene expression variation in six tissue types, the color represents gene expression levels. The number represents the amount of genes in each tissue. (**E**) IGV snapshots showing expressed tissue-specific genes with tissue-specific TE-derived ACRs.

ACRs have been reported to play vital roles in the regulation of gene expression in eukaryotic genomes by interacting with *trans*-acting factors [36,37]. However, the role of TE-derived ACRs in the regulation of rice gene transcription remains unclear. To address this, we calculated the genomic distributions of TEs and ACRs and found that all ACRs and non-TE-derived ACRs tended to be located in genic regions, especially in promoters. In contrast, TEs and TE-derived ACRs were distributed more in the distal intergenic regions and downstream of genes (Figure 3A), which is consistent with previous findings in mammals that TE-derived ACRs can act as potential enhancers [34]. We then divided all ACRs as either common (*n* = 70,624) or tissue-specific (*n* = 12,463) ACRs, according to the entropy values (Figure S8 and Table S2). Compared with common ACRs, tissue-specific ACRs were distributed more in the distal regions (Figure 3B). Moreover, we found that less tissue-specific ACRs were TE-derived (3.5%), including 1.3% of pACRs, 0.8% of dACRs, 0.5% of gACRs, and 0.9% of dnACRs (Figure 3B), while more TE-derived ACRs were found in the common ones (8.6%), these TE-derived tissue-specific ACRs were mainly enriched in *hAT*, *LINE*, *Gypsy* and other *LTR* retrotransposons, as compared to the whole genome background (Figure 3C). After calculating the fold-change in accessibility levels for tissue-specific ACRs in six tissue types, we found a similar variation between TE and non-TE-derived tissue-specific ACRs (Figure S9), suggesting that TE-derived ACRs can also be involved in tissue development like regular ACRs. To confirm this, we calculated the expression levels of TE-derived ACRs nearby genes in six tissues, and found that a subset of genes exhibited a similar trend to the changes in chromatin accessibility levels (Figure 3D). As shown in Figure 3E, some TE-derived tissue-specific ACRs were located at the upstream of subsets of functional tissue-specific genes. For example, a *MULE*-derived ACR was located in the promoter of a hydroquinone glucosyltransferase encoding gene (*LOC_Os03g44180*), preferentially expressed in root (RT); a *hAT* transposon related ACR was located near the TSS of *NL1* with higher expression levels in young panicle (YP), which is a GATA type transcription factor related to the panicle development [38]; a *Gypsy* retrotransposon-derived ACR was located in the upstream region of *OsBISAMT1*, a gene encoding S-adenosyl-L-methionine related to defense responses, which was specially expressed in young leaf (YL) [39].

Collectively, our results show that TE-derived ACRs may play vital roles in the regulation of tissue-specific gene expression, thereby being involved in the tissue development in rice.

### 2.4. Impacts of TEs on Chromatin Accessibility and Its Conservation

**Figure 4:**
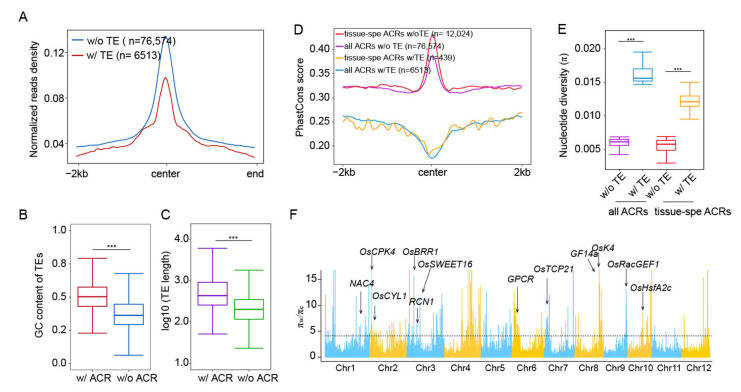
Genomic features and conservation of TE-derived ACRs. (**A**) Curve plot demonstrating normalized ATAC-seq read density across ±2 kb of the center of ACRs with (*w*/)/without (*w*/*o*) TEs in flag leaf (FL). (**B**) Boxplot showing the difference of GC content of TE (with/without ACRs). Significance test was determined using Wilcoxon rank sum test, *** *p* < 0.001. (**C**) Boxplot showing the difference of TE (with/without ACRs) length. Significance test was determined using Wilcoxon rank sum test, *** *p* < 0.001. (**D**) Averaged phastCons score across 4 kb regions centered by all ACRs and tissue-specific ACRs, with or without TEs. (**E**) Boxplot demonstrating mean π values around 2 kb region of ACRs with/without TEs. Significance test was determined using Wilcoxon rank sum test, *** *p* < 0.001. (**F**) Selection signals in the rice genome. The horizontal black dashed lines showing the genome-wide threshold for domestication sweeps (π_w_/π_c_ > 4.1). Functional genes overlapped with TE-derived ACRs under domestication selection were labeled in corresponding chromosomes.

To investigate how the presence of TEs affects chromatin accessibility, we plotted normalized ATAC-seq read counts across ±2 kb of the center of ACRs with and without TEs (Figure 4A and Figure S10), and found that non-TE-derived ACRs were more open than TE-derived ones. At the sequence level, we found that TEs containing ACRs had a longer length (Figure 4B). A similar trend was also observed in TEs superfamilies (Figure S11), and TEs containing ACRs had higher GC content, which facilitates nucleosome formation [40], as compared to inaccessible TEs (Figure 4C).

It is well known that the transposition activity of TEs potentially leads to various genomic mutations during evolution [41]. To explore the conservation of TE-derived ACRs during rice evolution, we compared the PhastCons score (representing conservation landscapes among 63 different plant species) for different types of ACRs [42], including the tissue-specific ACRs shown in Figure 3B. We found that TE-derived ACRs had much lower PhastCons scores than non-TE-derived ACRs, especially in the center regions of ACRs, and tissue-specific ACRs showed significantly higher PhastCons scores than all ACRs (Figure 4D). These results indicated that most of TE-derived ACRs were less conserved at the sequence level than the non-TE-derived ones and tended to be species-specific during plant evolution; in contrast, tissue-specific ACRs, exhibited a high conservation between different plant species, which may be caused by directional selection. We then calculated the π values, which can reflect the nucleotide diversity within different rice varieties, for the ACRs shown in Figure 4D, and found that TE-derived ACRs had higher π values, while tissue-specific ACRs had much lower π values than all ACRs (Figure 4E), indicating that conservation changes for different types of ACRs are highly associated with the presence of TE during rice domestication. Domestication usually leads to adaptive changes in rice. To determine whether TE-derived ACRs are also involved in domestication, we identified genomic regions with notable decreases in nucleotide diversity by comparing the π values between the wild (π_w_) and cultivated rice accessions (π_c_). According to π_w/_π_c_ values, we identified 187 putative domestication sweeps, containing 270 TE-derived ACRs (~4%) (Figure 4F). Indeed, we found that in these domestication regions, some genes essential for the rice development were associated with TE-derived ACRs, including flower development gene *OsK4* [43], hormone-related gene *OsCYL1* [44], and several disease/stress resistance genes (*OsCPK4, OsBRR1, RCN1, OsSWEET16, GPCR, OsTCP21, GF14a, OsRacGEF1, OsHsfA2c)* [45,46,47,48,49,50,51,52,53].

Taken together, these results indicate that TE-derived ACRs may have coevolved with nearby genes during artificial and natural selection, thereby functioning in the formation of favorable agronomic traits in rice.

### 2.5. Relationship between TE-Derived ACR around TSS and Duplicated Genes

**Figure 5:**
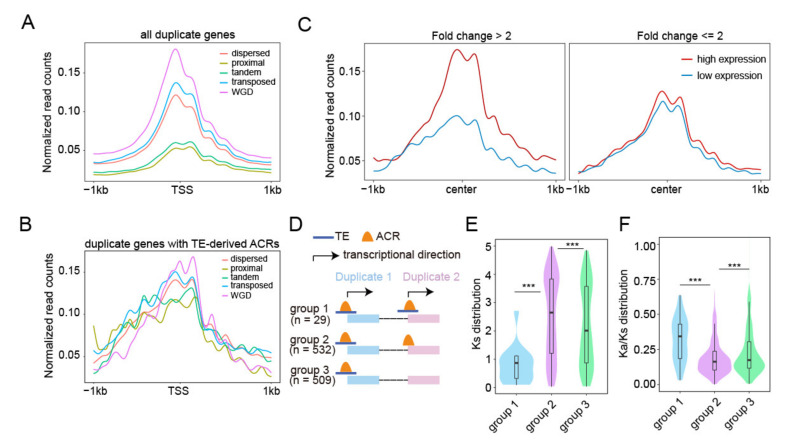
The chromatin accessibility for duplicated gene pairs. (**A**) Genome-wide normalized ATAC-seq read density of WGD, transposed, tandem, proximal, dispersed duplicated genes. (**B**) Normalized ATAC-seq read density of duplicated genes associated with TE-derive ACRs. (**C**) Correlation between chromatin accessibility changes and gene expression changes of duplicated gene pairs corresponding to TE-derive ACRs. All gene pairs were divided into two groups according to their expression differences: fold change > 2 and fold change <= 2, the chromatin accessibility variations showing greater differences in the fold change > 2 group. (**D**) Gene pairs with TE-derived ACRs around TSSs were divide into three subgroups. Only 29 pairs of duplicated genes had TE-derived ACRs around TSSs of both copies. (**E**,**F**) Comparisons of distributions of *Ks* (**E**) and *Ka/Ks* (**F**) between duplicated gene pairs in three groups are illustrated in (**D**). Significance test was determined using Wilcoxon rank sum test, *** *p* < 0.001.

Gene duplication is one of the major driving forces for genome complexity and gene subfunctionalization or neofunctionalization during plant polyploidization and evolution [54,55,56]. Growing evidence suggests that epigenetic mechanisms are involved in the regulation of duplicated gene transcription [57,58,59,60,61]. TEs have been found to affect the epigenetic status of duplicate genes [62], however, how TE-derived ACRs function in the regulation of duplicated genes in rice remains unclear. To answer this question, we chose ATAC-seq data from the stamen and pistil (SP) for analyses because it has the most ACRs among the six tissues. We plotted normalized ATAC-seq read counts across ±1 kb for all duplicated genes (Figure 5A and Table S3). We observed that differential read enrichment levels occurred among genes in different duplicate modes and a significant read abundance occurred around the TSSs (Figure 5A). Given that epigenetic features around the TSS usually play an important role in the regulation of gene expression, we specifically counted duplicated genes (*n* = 561) with TE-derived ACRs at ±500 bp of the TSS (Table S4) and observed a similar trend in reads enrichment between these duplicated genes and all genes shown in Figure 5A. Chromatin was more accessible for the WGD genes, whereas it was much less accessible for the proximal and tandem duplicated genes (Figure 5B), and the variation between different duplicate modes was smaller in genes related to TE-derived ACRs, which may cause by the fewer number of related genes and lower accessibility of TE-derived ACRs. Since these 561 duplicated genes corresponded to 1070 duplicated gene pairs in the rice genome (Table S5), we compared the expression level and chromatin accessibility of these gene pairs and found that the change in chromatin accessibility exhibited a positive correlation with the expression change of gene pairs (Figure 5C), indicating that the chromatin dynamics of TE-derived ACRs are involved in the regulation of gene pair expression.

TEs can act as potential novel regulatory sequences in the host genome, and TE inserted near genes can be selected during evolution [2]. To identify TE-related regulatory sequences of duplicated genes, we divided all duplicated gene pairs with TE-derived ACRs around the TSSs into three subgroups: subgroup 1, duplicated gene pairs with both copies of TE-related ACRs around the TSSs (*n* = 29); subgroup 2, duplicated gene pairs with TE-related ACRs around the TSSs for one copy and regular ACR for the other copy (*n* = 532); subgroup 3, duplicated gene pairs with only one copy having TE-related ACRs around the TSSs (*n* = 509) (Figure 5D). After conducting novel motif enrichment assays, we found that the majority of motifs overrepresented in ACRs were “G”- or “A”-rich sequences. In particular, we found that the motif for the binding of the TCP transcription factor was overrepresented in TE-related ACRs instead of regular ACRs (Figure S12), indicating that TE insertion can introduce new regulatory DNA sequences, which may be involved in the modulation of gene pair expression. Furthermore, we examined the differential distributions of *Ks* (synonymous substitutions per synonymous site) among duplicated gene pairs and observed the descending order of *Ks* distribution changes among duplicated genes in three subgroups: subgroup 1 < subgroup 3 < subgroup 2 (Figure 5E), indicating a distinct TE-derived ACRs distribution between younger (lower *Ks* value) and older gene duplicates (higher *Ks* value). To assess whether genes with the absence/presence of TE-related ACRs around TSSs were subjected to different intensities of selective constraints, we calculated *Ka* (nonsynonymous substitutions per nonsynonymous site)*/Ks* values for gene pairs, and found that most of the *Ka/Ks* were less than 1 (Figure 5F). For subgroups of gene pairs, we found that subgroups 1 and 2 had the highest and lowest mean levels of *Ka/Ks*, respectively (Figure 5F).

These results suggest that TE-derived ACRs related gene pairs are primarily subjected to purifying selection, and a combination of TEs and ACRs is associated with the strength of the selection pressure.

## 3. Discussion

TEs can induce a wide range of genomic alterations, including chromosome rearrangement, epigenetic regulation, and insertion mutagenesis [63], resulting in genetic diversity and genome evolution. Various epigenetic mechanisms have been found to regulate TE activity in plants [64]. The footprint of TE could regulate gene expression by changes in epigenetic modifications [2,65]. The function of TEs in the regulation of gene transcription can be mediated by the formation of open chromatin in maize [66] and mouse [31]. TEs account for up to 40% of the rice genome and its superfamilies have already been well characterized [63]. However, the mechanism by which TEs regulate gene transcription in rice remains understudied. Our study revealed several discoveries by associating TEs with open chromatin and epigenetic modifications.

Global identification of ACRs helps to mine functional CREs like promoters, enhancers, et al. Characterization of TE-derived ACRs helps to identify CREs associated with TEs, thereby advancing understanding of roles of TEs in the regulation of gene expression. Accumulating evidence shows variations in the formation of TE-derived CREs among different organisms. For instance, nearly 30% of TEs containing active elements, with 45% and 20% of them belonging to LTR retrotransposons and DNA transposons, respectively, were found in the classic model organism *D. melanogaster* [67]; 25% of TE-derived CREs were found in humans, and of which the LTRs contain almost all of the TFBSs [68,69]; About 20% of open chromatin regions associated with TEs were found in mouse, which mainly consist of LTR and SINE retrotransposons [31]. Similarly, TE-derived ACRs have also been reported in plants. Over 57% and 26.7% TE-derived DHSs were found to be associated with LTRs, and DNA transposons in maize, respectively [30]. Our study showed that ~8% ACRs had more than 50% sequence covered by TEs in rice. It is worth noting that our analyses cannot distinguish if TE-derived ACRs are from TEs carrying ACRs or insertion of TEs into pre-existing ACRs within the genome. After a closer examination, we found that 45.5% of these ACRs were fully covered by TEs, which are most likely associated with TE-derived ACRs; the remaining ones were partially (50–99%) covered by TEs, which could be further divided into two groups, the length of TEs is shorter than that of ACRs, and the TEs overlapping with one edge of ACRs. Technically, it is not easy (nearly impossible) to distinguish the two distinct cases of TE insertion. In our opinion, our analyses indicate that as least parts of TEs if not all have the potentials to form ACRs, which has already been reported in other species [34]. Comprehensive comparisons of TEs and ATAC-seq data between different genomes help to identify polymorphic TE- and shared TE-derived ACRs between species. Our study showed that DNA transposons contributed to most of the TE-derived ACRs in rice, which is different from the findings reported in aforementioned organisms. This is possibly caused by characteristics of rice genome. The copy number of DNA transposons far exceed that of retrotransposons, while most retrotransposons are longer and prone to locate in distal regions of genes. The insertion of some short DNA transposons could influence the activities of nearby genes. We found the majority of retrotransposons contribute to ACRs in rice were *LTR_Copia*, it was reported some solo-LTRs tend to keep TF-specific CREs [70], indicating that functions of these LTRs in the regulation of gene expression can be mediated through distal interactions between CREs and TFs or *trans*-acting factors. Variation of TE-derived CREs could reflect differentiation of TE activities among different organisms during genome evolution.

The degree of chromatin openness correlated negatively with the density. This is possibly caused by the overall hypermethylation of TEs in plant genomes [71,72,73], while hypomethylation favors the formation of open chromatin [35]. Consistent with findings about TEs sequestered far from genes derived by purifying selection and genetic drift during evolution [74], a much higher normalized TE number was observed around dACRs than around pACRs in rice (Figure 2A). This provided evidence showing TE subtype-dependent effects on the formation of ACRs, which is possibly mediated by changes in local DNA methylation. A similar finding was reported in mouse [34], indicating that the differential functions of TE superfamilies in ACRs formation are conserved between plants and mammals.

CREs embedded in open chromatin regions can be activated in a tissue-specific manner [27,31,75,76]. Our study provided evidence that the connections between TEs and tissue development can be mediated by the formation of TE-derived ACRs that regulate tissue-specific gene expression. For example, the known functional gene *NL1* and *OsBISAMT1* were found to have tissue-specific TE-derived ACRs in their promoters (Figure 3E). Similar findings have been reported in humans [77,78,79]. This implies that the chromatin status in TE-derived ACRs could be dynamic in order to temporally regulate nearby gene expression during tissue development or in response to external environmental cues. In addition, if possible, analyses of TEs among different tissues of a single plant or between individual plants help to identify tissue or individual plant related polymorphic TE insertions, which could help to address variations of TE functions at the population level or a tissue wide.

Chromatin accessibility is positively correlated with the conservation of accessible chromatin regions [80]. Our study showed that TE-derived ACRs were involved in rice domestication, as evidenced by the following: TE-derived ACRs were more accessible than ACRs without TEs; TE-derived ACRs exhibited low conservation and high nucleotide diversity; and more importantly, domestication sweep regions of rice had some agronomic trait-related genes with TE-derived ACRs (Figure 4), indicating that these ACRs-related TE insertions in rice may favor co-selection and domestication of key agronomic traits as opposed to having detrimental impacts on the genome. The selection is also related to the epigenetic status of the TEs. For instance, hypermethylated TEs that insert nearby genes in *Arabidopsis* are strongly selected against, ultimately resulting in elimination of related TEs [2].

Expression divergence of duplicated genes results in the neo- or subfunctionalization of duplicated genes [81], which can be regulated by changes in epigenetic modifications, such as DNA methylation and histone modifications [57,58,60]. It has been reported that TEs can affect the evolutionary divergence of duplicated genes through changes in epigenetic status [62]. Our study indicated that TE-derived ACRs were involved in the divergence of duplicated gene pairs through the regulation of their expression. This was evidenced as follows: divergence of TE-derived ACRs was positively correlated with the expression changes of duplicated genes, which could be mediated by divergence of the underlying regulatory sequences [82]; gene pairs with both copies having TE-derived ACRs tended to be younger than others; and duplicated gene pairs having a TE-derived ACR around the TSSs for one copy and a regular ACR for the other copy (subgroup 2) experienced stronger purifying selection than those with both copies having TE-derived ACRs around the TSSs (subgroup1) (Figure 5). Young gene pairs have similar TE environments [62]. The TE insertions affect the selection strength of nearby genes [83,84]. TE-free genes have stronger purifying selection than TE-rich genes [84], indicating that the presence of TEs has a potential impact on the selection of subsets of genes.

## 4. Methods

### 4.1. Processing of Public RNA-Seq, ATAC-Seq and ChIP-Seq Data

Public RNA-seq and ATAC-seq data with high quality from six rice tissues [33], and ChIP-seq data from young leaves [28] of rice variety Zhenshan97 were used in this study. Fastp (v0.21.0) [85] was used to filter the raw reads. Any reads with low-quality values (Q < 25) and short lengths (<50 bp) were excluded from further analyses. Clean reads of both data were mapped to the rice MSU7.0 reference genome as the previous study did [33] using Hisat2 (v2.1.0) [86] and Bowtie2 (v2.2.5) aligner [87], respectively. For RNA-seq data, featureCounts [88] was used to count the reads mapped to the rice genes. A custom R script was used to calculate the fragments per kilobase million (FPKM) values, representing the expression level of each gene.

For the ATAC-seq and ChIP-seq data, PCR duplicates were removed using Picard MarkDuplicates (http://broadinstitute.github.io/picard/, accessed on 22 March 2022). Samtools (v1.9) [89] was used to remove aligned reads with a mapping quality (MapQ) less than 30, followed by the conversion of bam files to bigwig files using deeptools (v3.1.3) [90] based on the fragments per kilobase per million reads mapped (RPKM) normalization method. The read distributions of both datasets across the rice genome were visualized using IGV (v2.4.13) [91].

### 4.2. BS-Seq Data Analyses

Public BS-seq data generated from young leaves of Zhenshan97 were used for the DNA methylation assay [28]. The raw paired-end reads were filtered using fastp (v0.21.0) [85] with the following parameters: lengthrequired = 50 –q 15. Bismark (v0.23.0) [92] was used for clean read mapping with the default parameters. After removing PCR bias using the deduplicate_bismark module, all uniquely mapped reads were retained for further analyses. The extent of methylation of each cytosine site was extracted using the bismark_methylation_extractor module. Only cytosine sites covered by at least five reads were retained for the methylation assay. CGmaptools (v0.1.2) [93] was used to convert cytosine methylation reports to CGmap formats. A custom Python script was used to calculate the methylation levels for the designated genomic regions.

### 4.3. Association Analyses of ACRs with Other Genomic Loci

MACS2 software (v2.1.4) [94] was used to define ACRs with the following parameters: −g 3.73134 × 10^8^ --no model --shift -50 --extsize 100 --q 0.01. Bedtools (v2.29.0) [95] was used to correlate the ACRs with other genomic loci. ACR-related genes represent genes (from 2000 bp upstream of the TSS to 2000 bp downstream of the TTS) overlapping ACRs. The TE annotation file was obtained following the published pipeline [96] with rice repeat library (rice7.0.0.liban) downloading from https://github.com/oushujun/riceTElib (accessed on 20 May 2022). ACRs with more than 50% of their length covered by a TE sequence were defined as TE-derived ACRs, and the corresponding TEs were also considered as ACR-related TEs. A custom Python script was used to calculate the normalized ATAC read counts within the designated genomic regions and genes.

### 4.4. Identification of Tissue Specific ACRs

A total of 83,087 ACRs were obtained by merging all ACRs in each of the six rice tissues according to their genomic coordinates, using the merge function from bedtools (v2.29.0) [95]. The ATAC reads from six tissues located within these ACRs were counted and normalized to reads per million mapped reads (RPM). The RPM value of each ACR across the six tissues was used to identify the tissue specificity. By using the Shannon entropy method [97,98], entropy values of all ACRs were arranged in an ascending order and the top 15% of ACRs were considered as tissue-specific ACRs.

### 4.5. Estimation of the PhastCons Scores and π Values for ACRs

The evolutionary conservation of ACRs was measured using phastCons conservation scores downloaded from PlantRegMap (http://plantregmap.gao-lab.org/cis-map.php, accessed on 8 June 2022), which were calculated by multiple genome alignments of 63 different plants [42]. Vcftools (version 0.1.16) [97] was used to calculate π values for the cultivated and wild rice groups. The relative vcf format files and resequencing data were downloaded from the Rice SNP-Seek database (https://snp-seek.irri.org/_download.zul, accessed on 8 June 2022). For the wild rice, the resequencing data of 5 *O. rufipogon* plants were obtained from the public literatures [99]. For identification of domestication sweeps, π values were calculated using 100 kb windows, and regions with the top 5% π ratio (π_w_/π_c_) were set as thresholds for putative domestication sweeps, as described in the previous study [100].

### 4.6. Identification of Rice Duplicated Genes and Calculation of Ka/Ks Values

The different modes of duplicated genes were identified using the *DupGen_finder* pipeline (https://github.com/qiao-xin/DupGen finder, accessed on 10 June 2022) [101]. Briefly, the protein sequences annotated by the Rice Genome Annotation Project (http://rice.uga.edu, accessed on 10 June 2022) were blasted against themselves with an E-value < 1 × 10^−10^. The collinear blocks and syntenic gene pairs were identified using MCScanX [102] with default parameters. The “duplicate_ gene_ classifier” tools in MCScanX were used to classify genes into different duplicate modes. The Perl script DupGen_finder.pl in *DupGen_finder* was used to match duplicated gene pairs, and the transposed duplicated gene pairs were detected using the *Zea mays* genome as their outgroups. *Ka*, *Ks* and *Ka/Ks* values of each duplicated gene pair were calculated following the public pipeline on GitHub (https://github.com/qiao-xin/Scripts_for_GB, accessed on 10 June 2022). *Ks* values >  5.0 were excluded from further analyses because of the saturated substitutions at synonymous sites [103]. Gene pairs with *p* value > 0.05 were also removed.

### 4.7. Motif Enrichment Analyses

The sequences of designated ACRs were used to identify motifs using the MEME-ChIP module of the MEME Suite Programs (https://meme-suite.org/meme/tools/meme-chip, accessed on 12 June 2022) [104,105], with DAP motifs [106] as known motif datasets. Motifs with E values less than 0.01 were defined as significantly enriched motifs.

## Data Availability

Illumina sequence reads of ATAC-seq and RNA-seq of the rice six tissues are available in the NCBI Sequence Read Archive database under accession number PRJNA705005, the raw data of ChIP-seq from rice young leaves are available from NCBI under accession number SRR10751569/SRR10751570 (H3K27me3), SRR10751567/SRR10751568 (H3K27ac), SRR10751565/SRR10751566 (H3K4me3), respectively. The BS-seq data from rice young leaves are available from NCBI under accession number SRR10763656.

## Supplementary Materials

Supplementary Materials can be found at https://www.mdpi.com/article/10.3390/ijms23168947/s1.
