# Supplementary material for: Characterization of Transposon-Derived Accessible Chromatin Regions in Rice (Oryza Sativa)
Source: Int J Mol Sci. 2022 Aug 11;23(16):8947. doi: 10.3390/ijms23168947 (PMC9408979; doi:10.3390/ijms23168947)
Supplement: Supplementary file 1 [file ijms-23-08947-s001.zip › Supplementary Files/Supplementary Figures.pptx]

## Slide 1
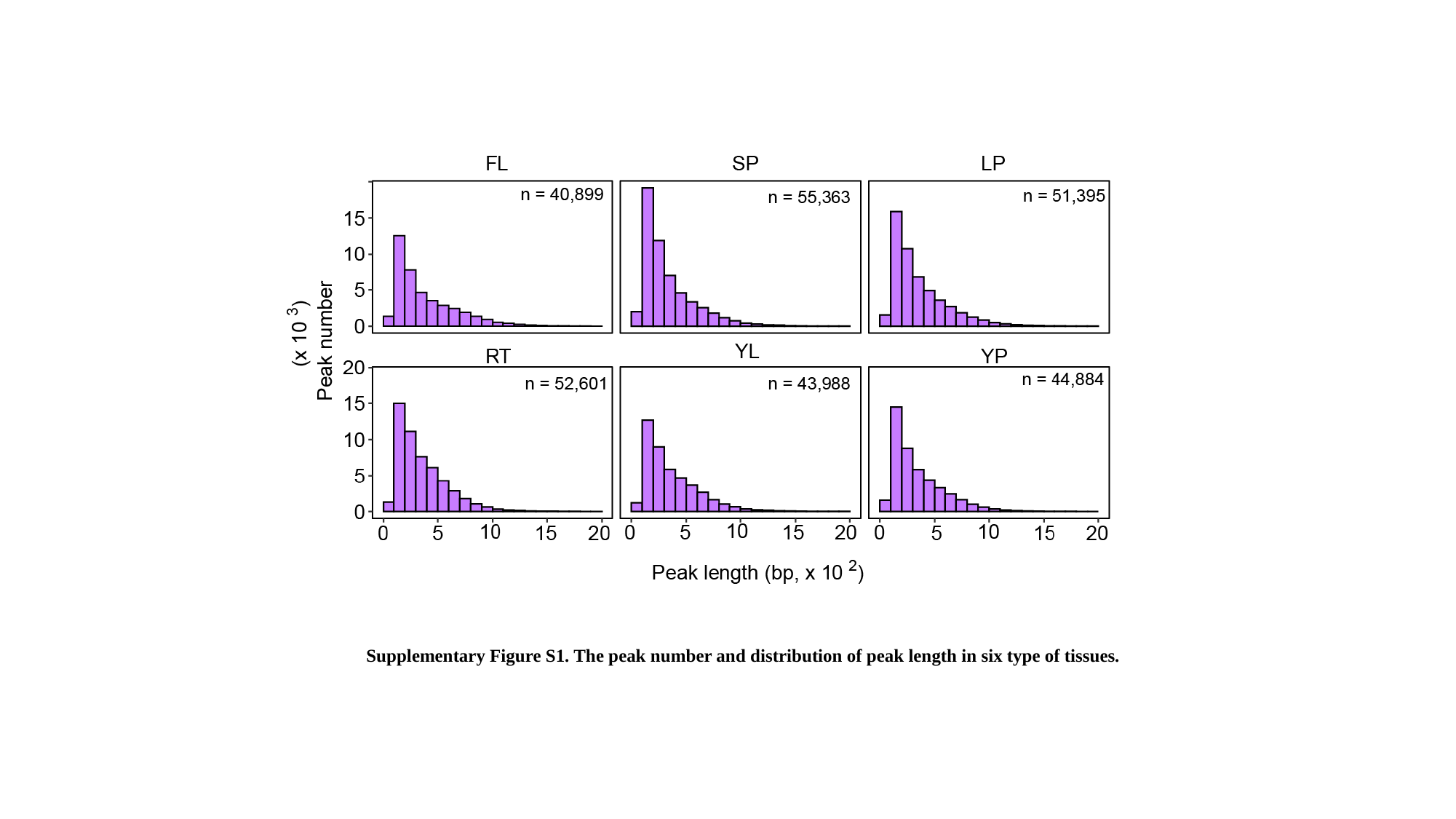

Supplementary Figure S1. The peak number and distribution of peak length in six type of tissues.

## Slide 2
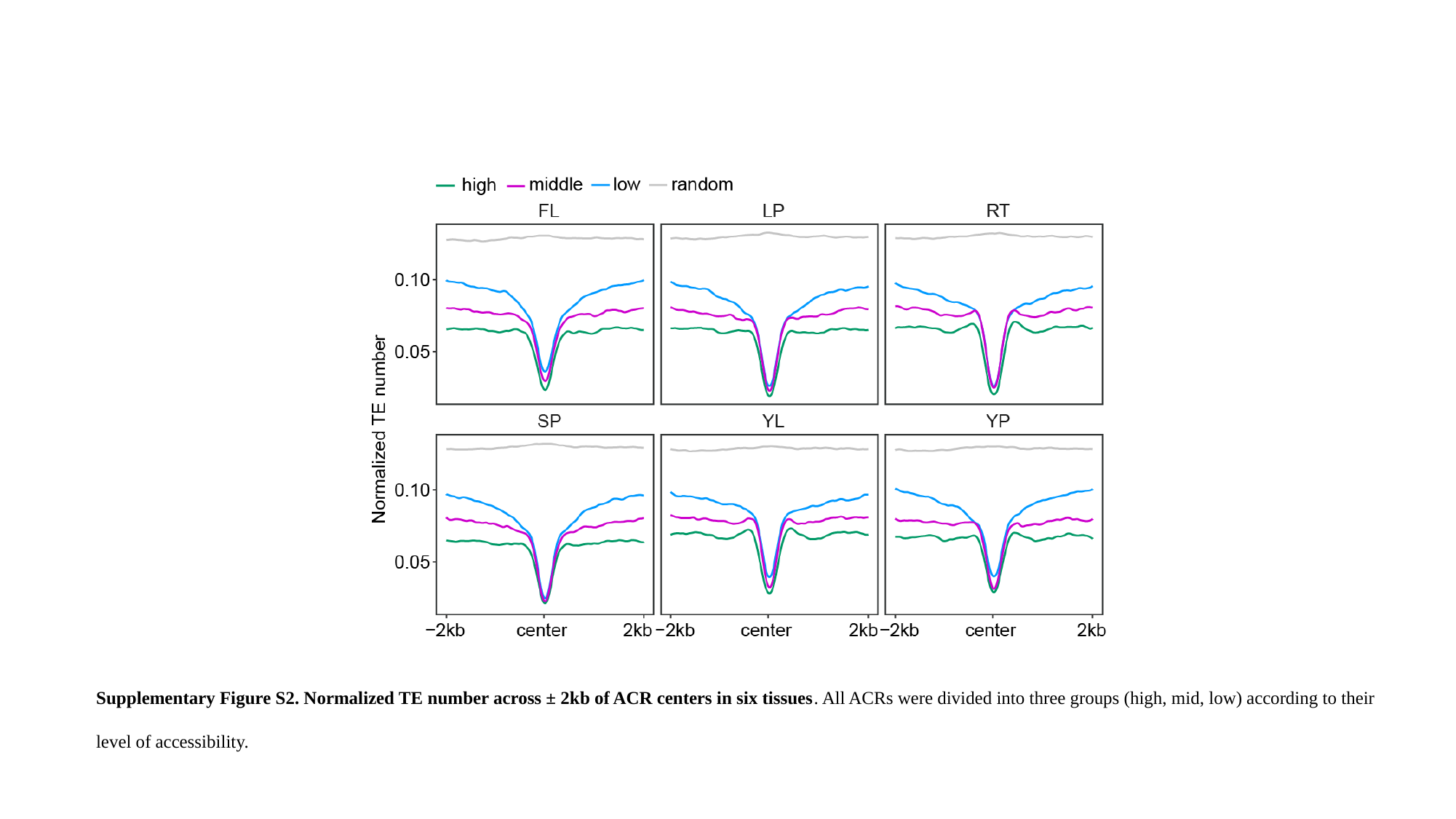

Supplementary Figure S2. Normalized TE number across ± 2kb of ACR centers in six tissues. All ACRs were divided into three groups (high, mid, low) according to their level of accessibility.

## Slide 3
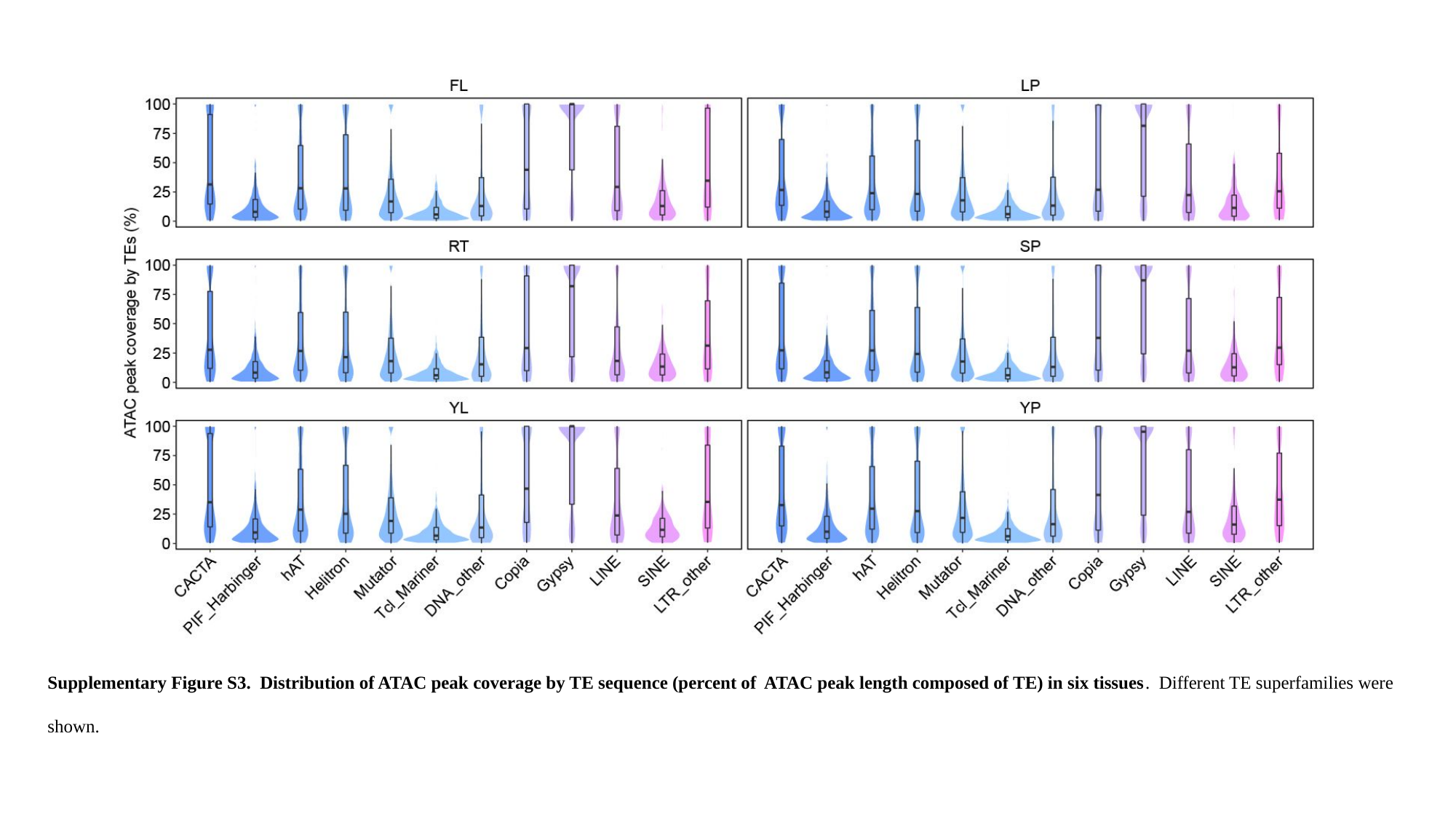

Supplementary Figure S3. Distribution of ATAC peak coverage by TE sequence (percent of ATAC peak length composed of TE) in six tissues. Different TE superfamilies were shown.

## Slide 4
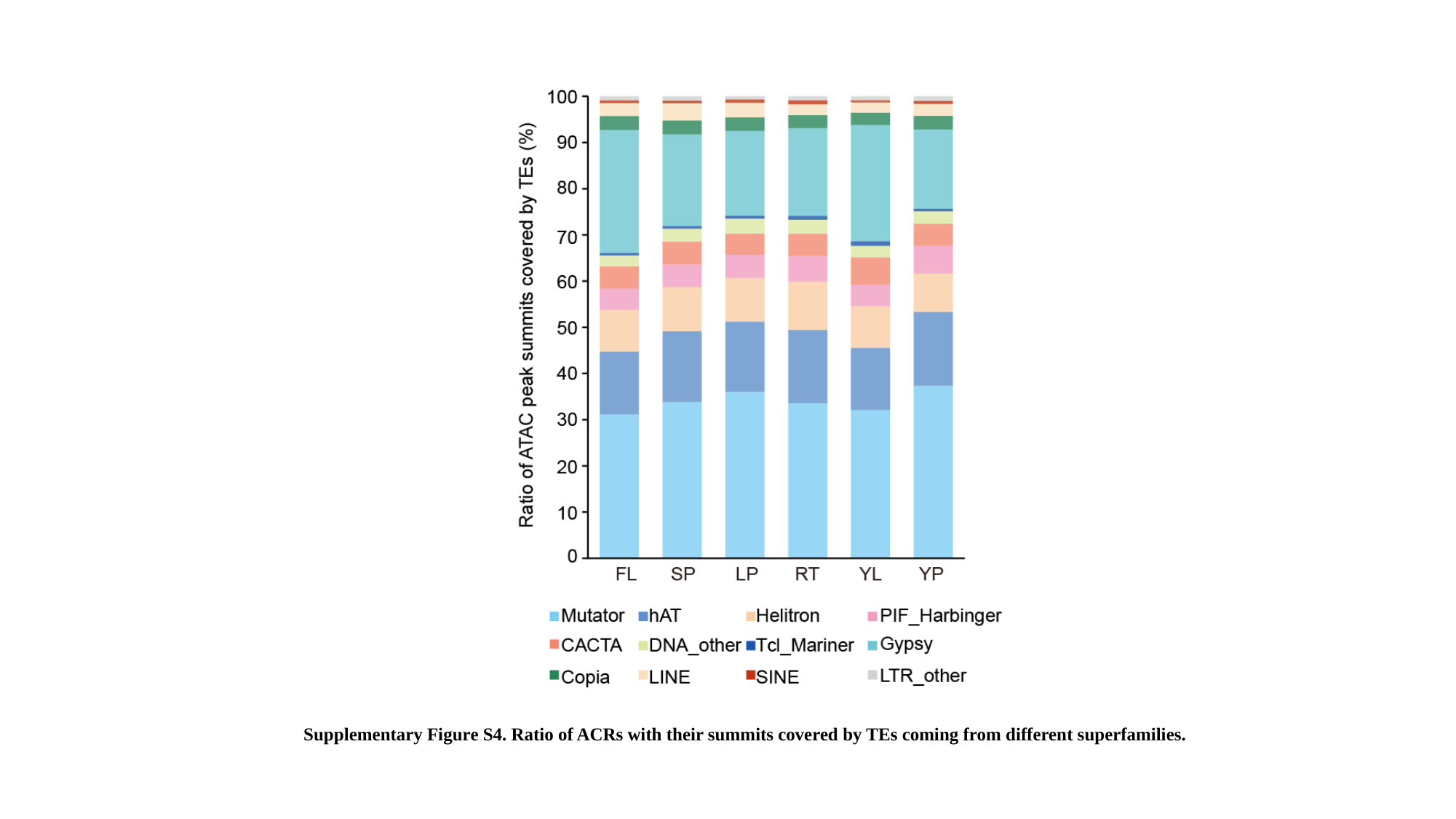

Supplementary Figure S4. Ratio of ACRs with their summits covered by TEs coming from different superfamilies.

## Slide 5
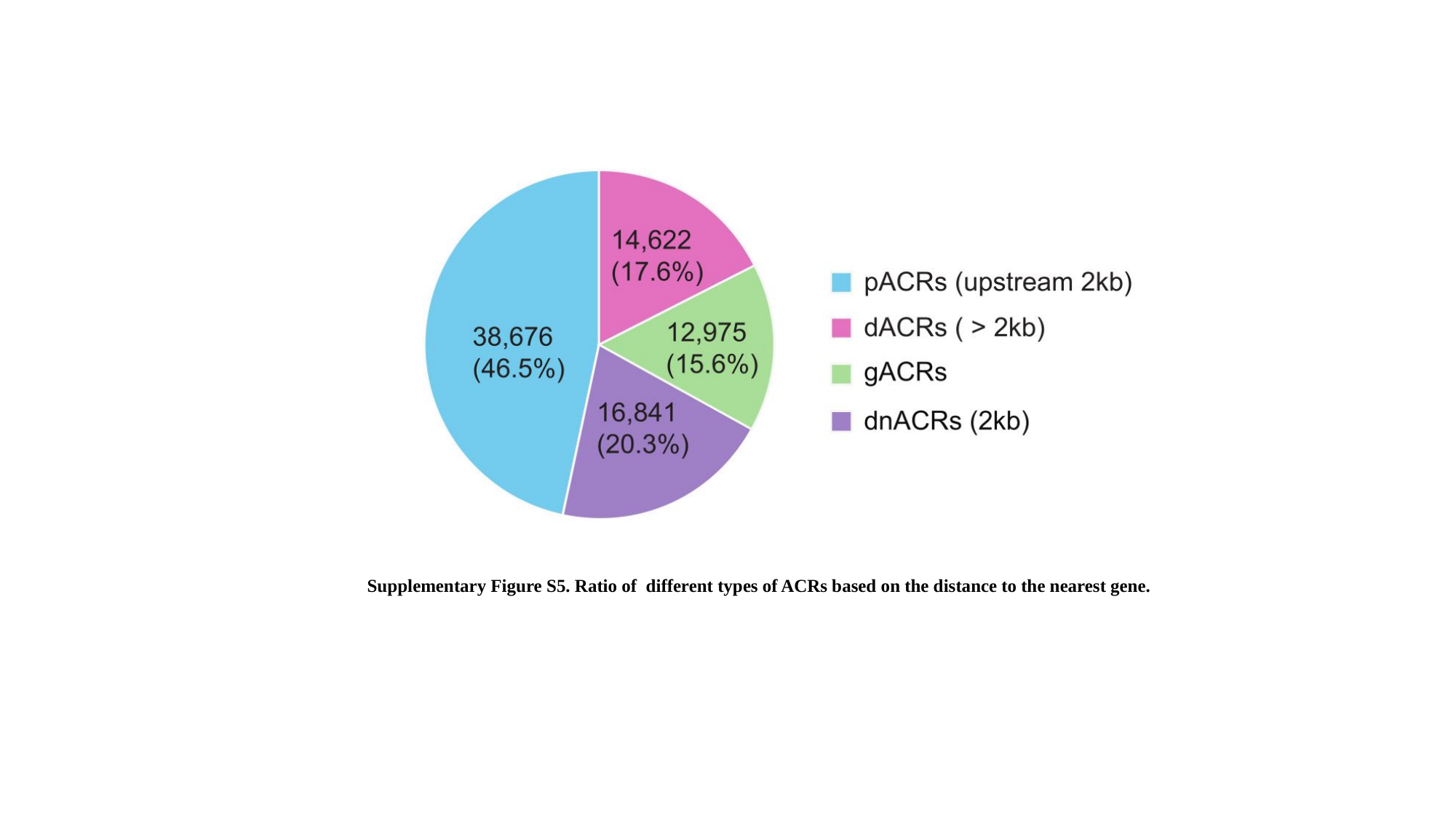

Supplementary Figure S5. Ratio of different types of ACRs based on the distance to the nearest gene.

## Slide 6
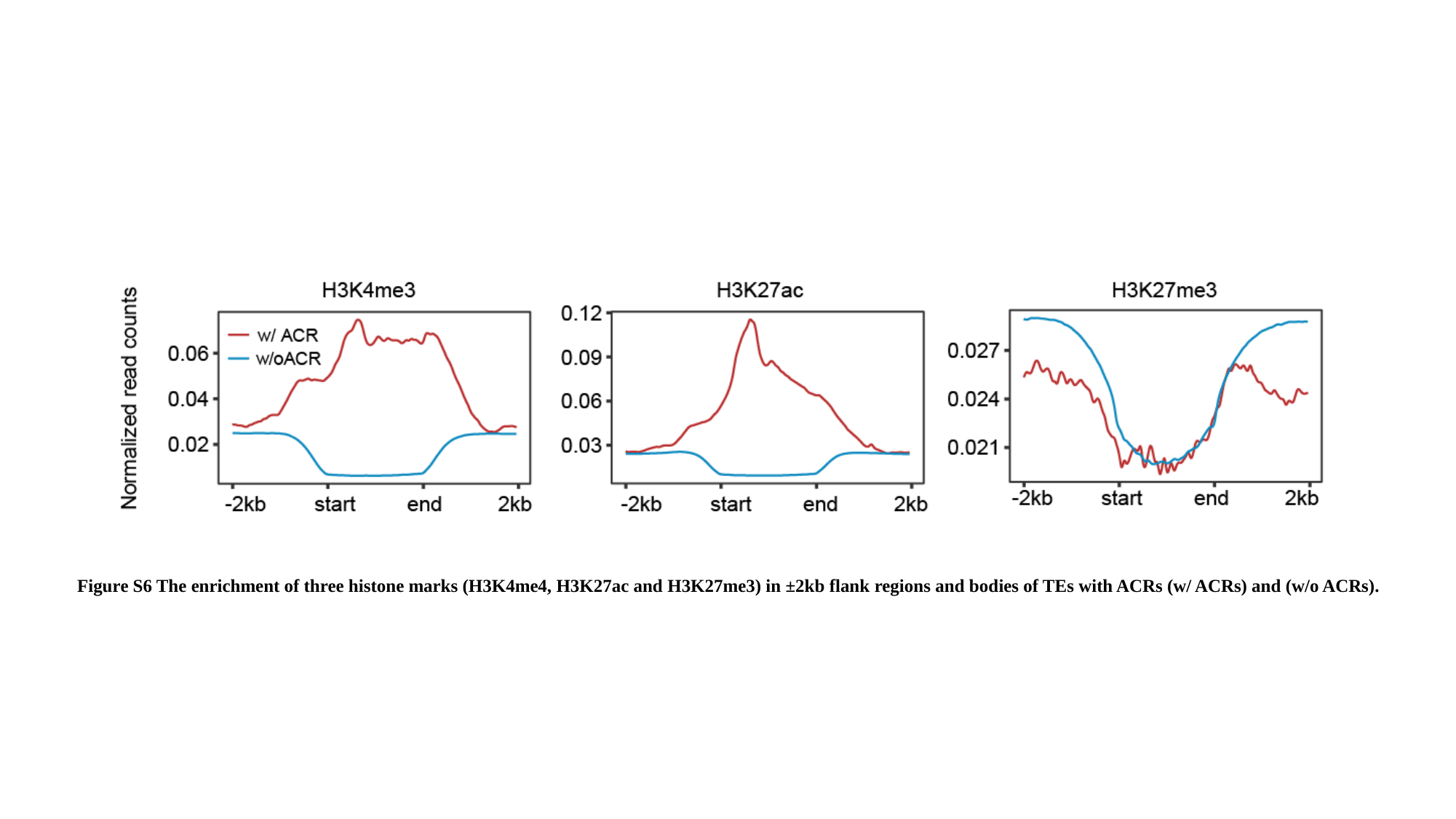

Figure S6 The enrichment of three histone marks (H3K4me4, H3K27ac and H3K27me3) in ±2kb flank regions and bodies of TEs with ACRs (w/ ACRs) and (w/o ACRs).

## Slide 7
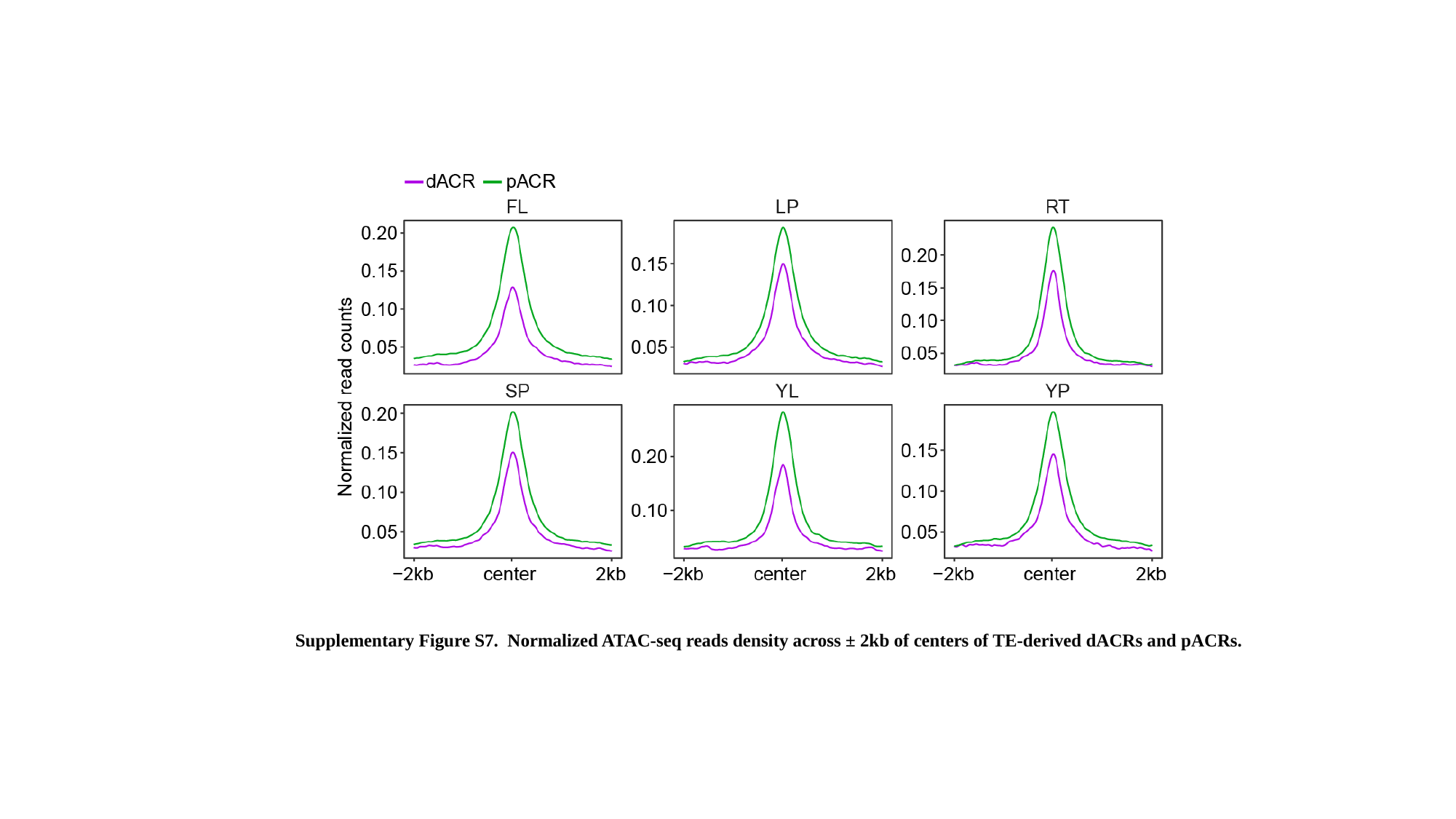

Supplementary Figure S7. Normalized ATAC-seq reads density across ± 2kb of centers of TE-derived dACRs and pACRs.

## Slide 8
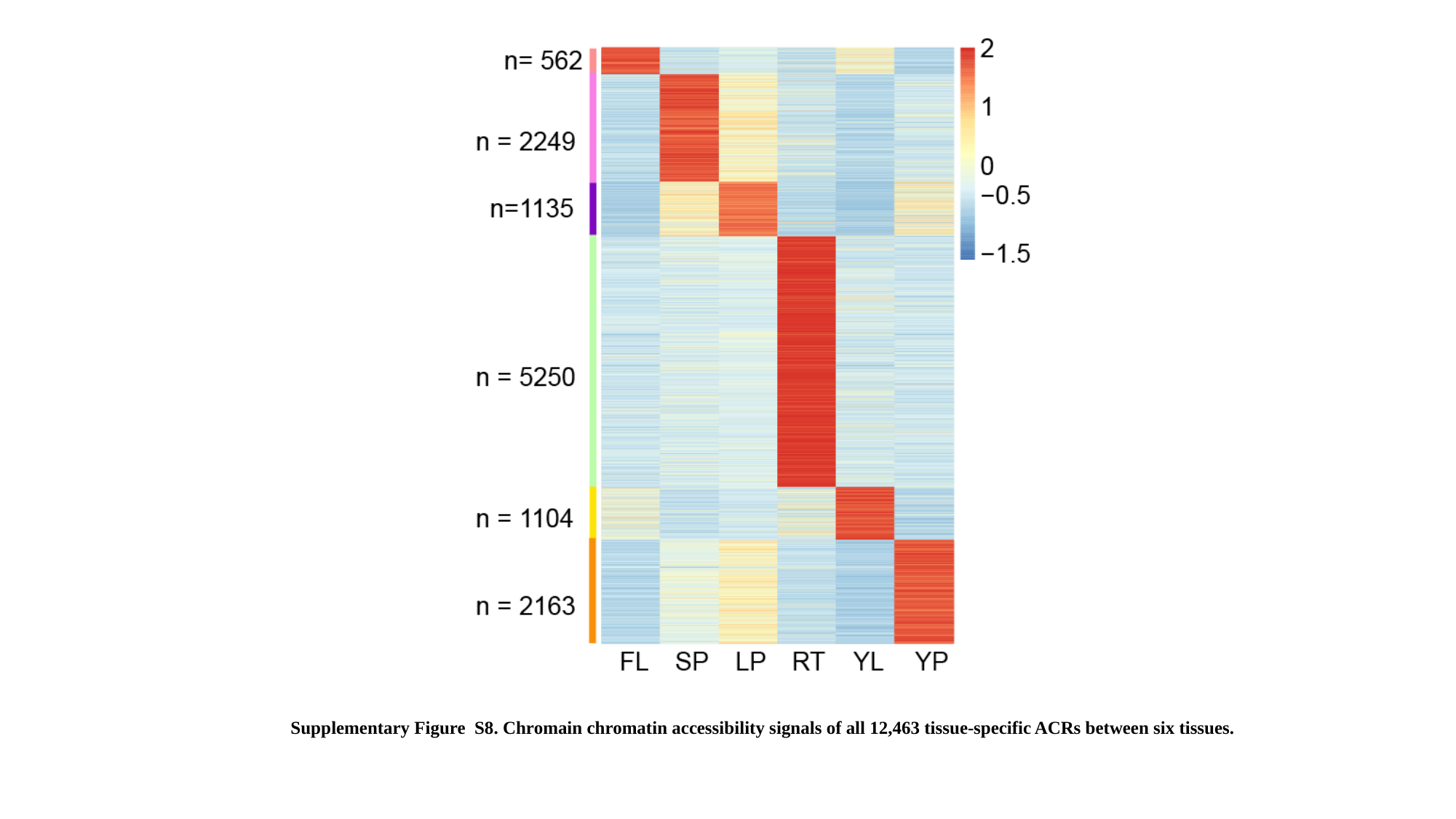

Supplementary Figure S8. Chromain chromatin accessibility signals of all 12,463 tissue-specific ACRs between six tissues.

## Slide 9
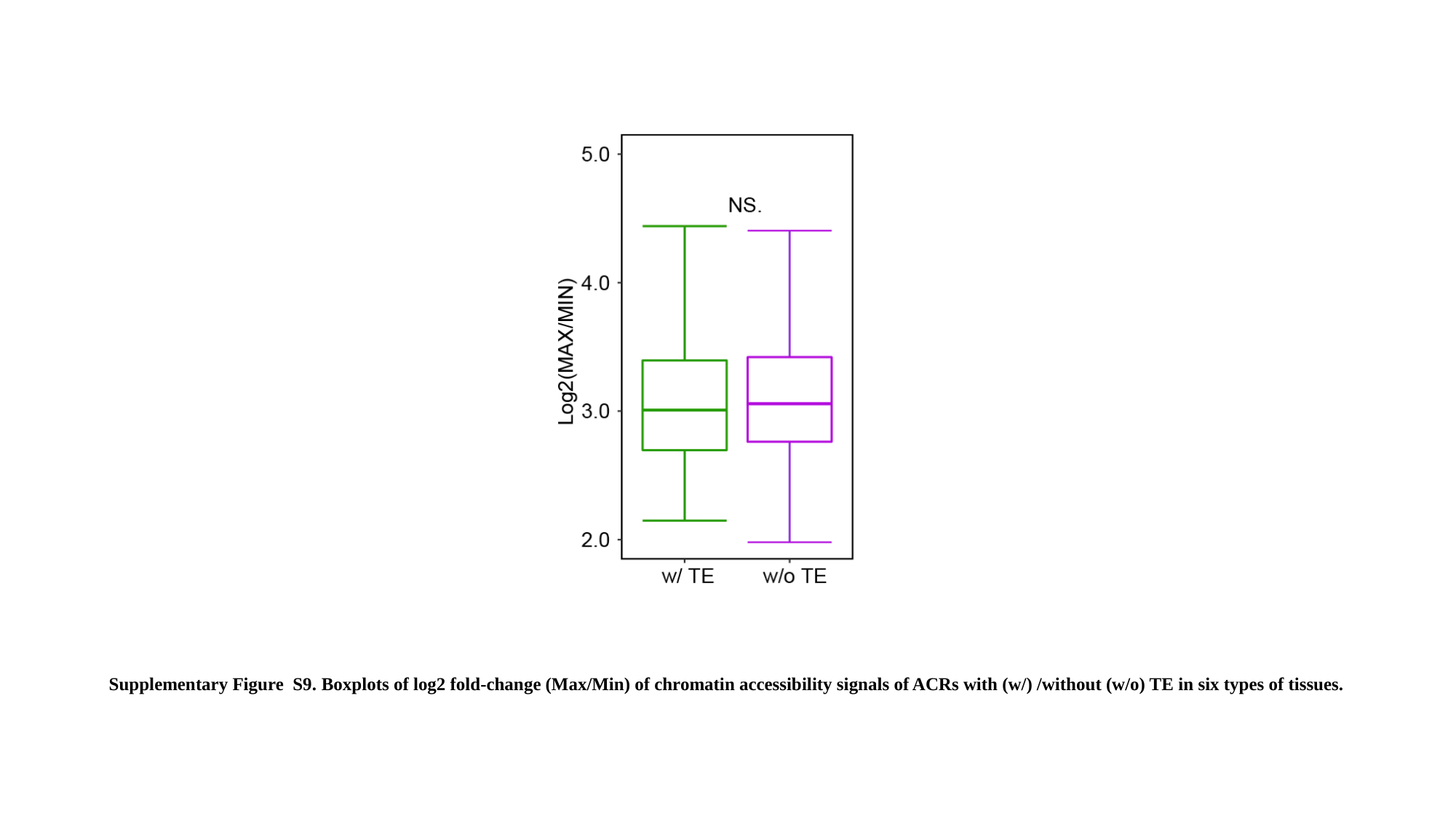

Supplementary Figure S9. Boxplots of log2 fold-change (Max/Min) of chromatin accessibility signals of ACRs with (w/) /without (w/o) TE in six types of tissues.

## Slide 10
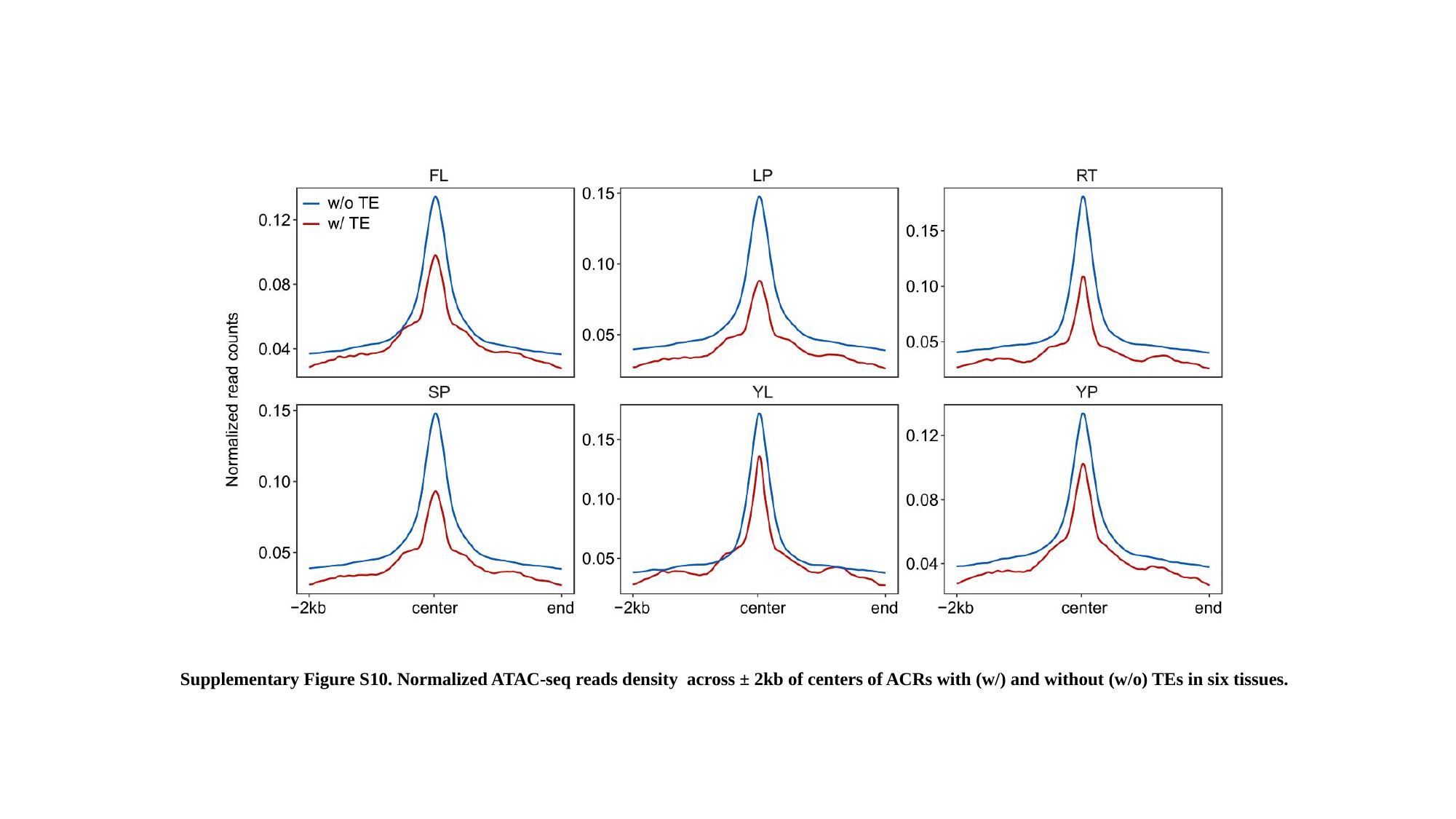

Supplementary Figure S10. Normalized ATAC-seq reads density across ± 2kb of centers of ACRs with (w/) and without (w/o) TEs in six tissues.

## Slide 11
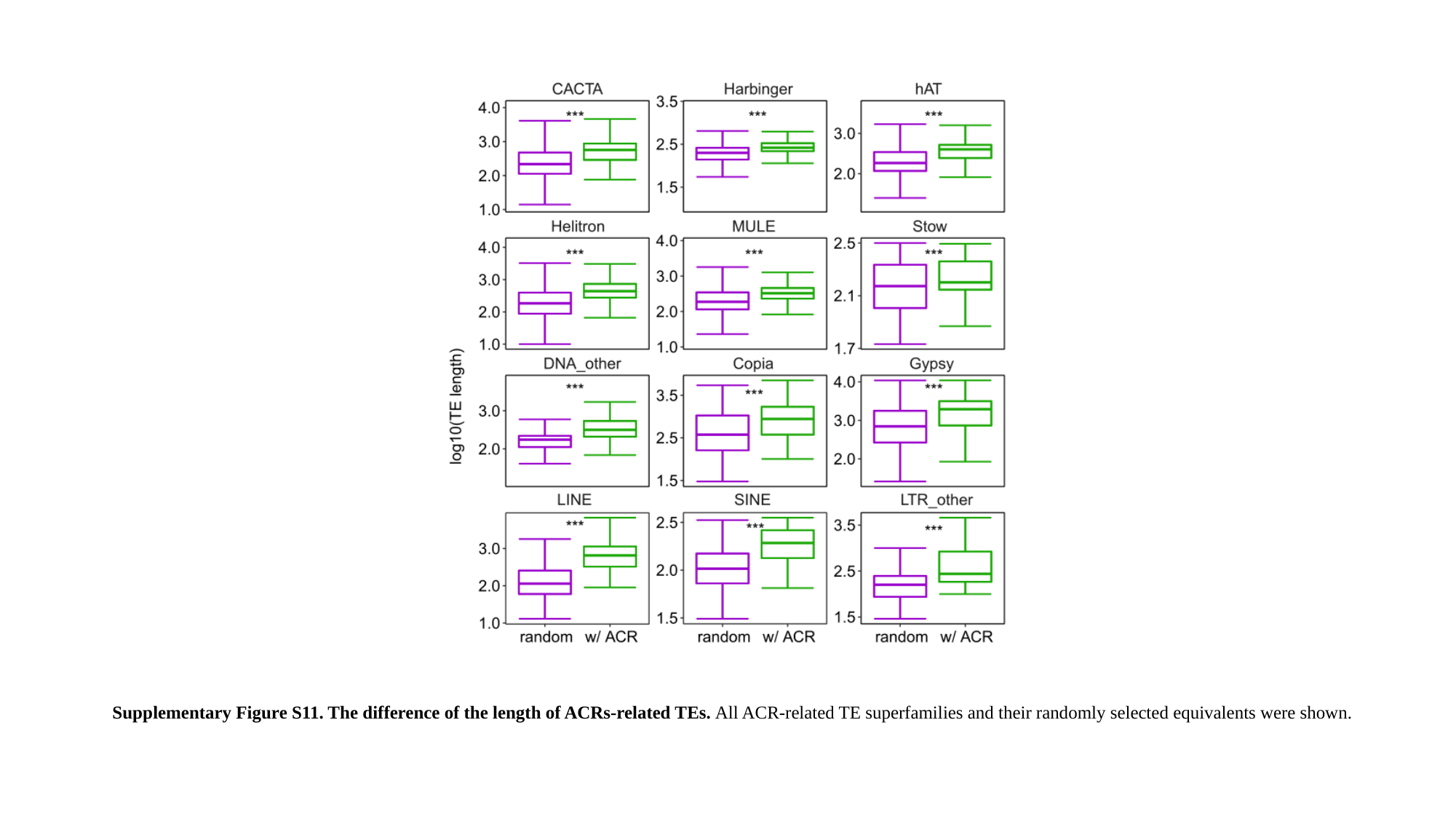

Supplementary Figure S11. The difference of the length of ACRs-related TEs. All ACR-related TE superfamilies and their randomly selected equivalents were shown.

## Slide 12
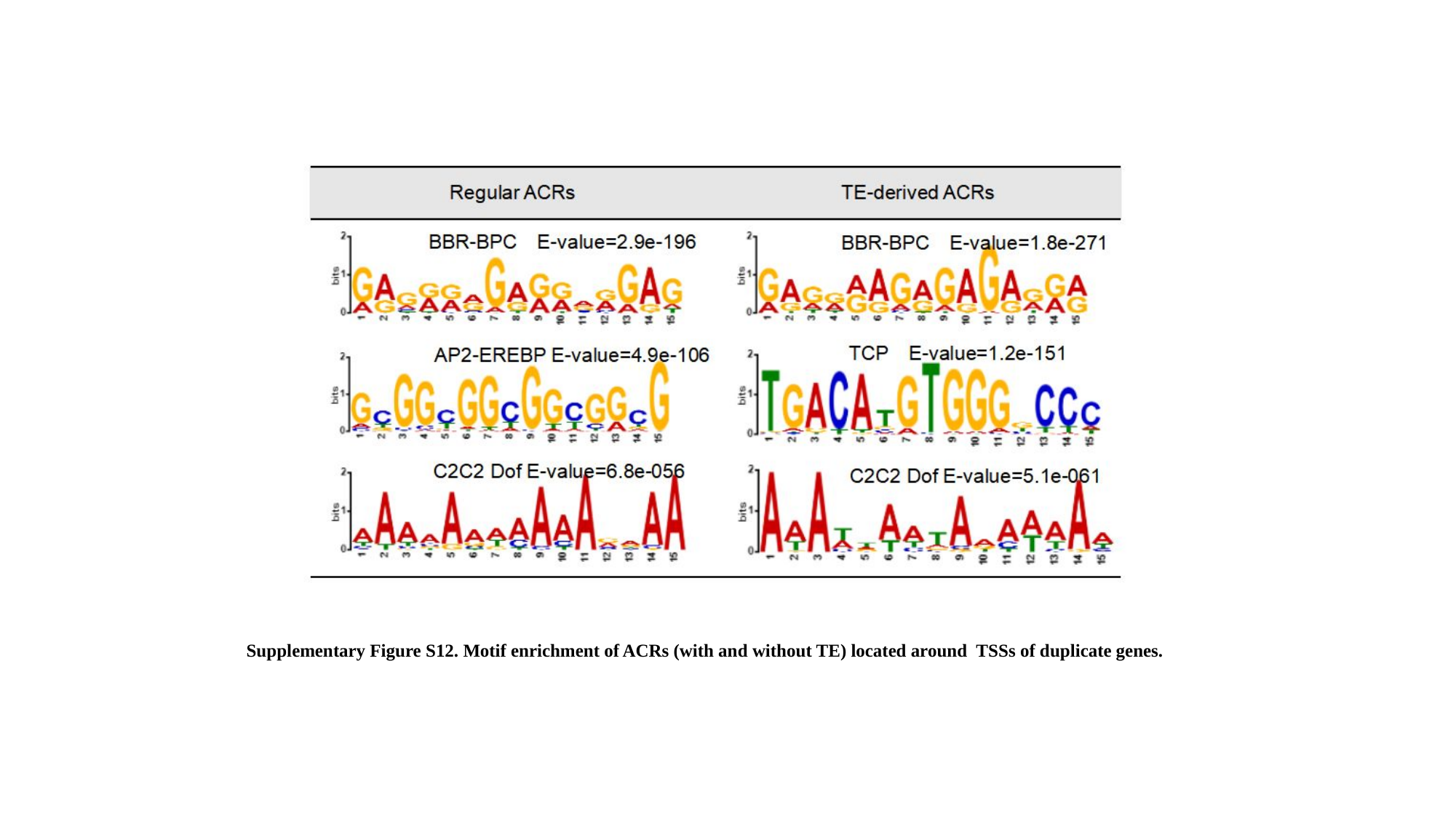

Supplementary Figure S12. Motif enrichment of ACRs (with and without TE) located around TSSs of duplicate genes.
